# Infection and Transmission of SARS-CoV-2 B.1.617.2 Lineage (Delta Variant) among Fully Vaccinated Individuals

**DOI:** 10.1128/spectrum.00563-22

**Published:** 2022-09-27

**Authors:** Leonardo C. Caserta, Mathias Martins, Brittany Cronk, Renee Anderson, Harry Eldridge, Deidre Gallow, Frank Kruppa, Elizabeth Plocharczyk, Diego G. Diel

**Affiliations:** a Department of Population Medicine and Diagnostic Sciences, Animal Health Diagnostic Center College of Veterinary Medicine, Cornell University, Ithaca, New York, USA; b Cayuga Medical Center, Cayuga Health Systems, Ithaca, New York, USA; c Tompkins County Health Department, Ithaca, New York, USA; University of Georgia

**Keywords:** B.1.617.2, breakthrough infection, COVID-19, Delta variant, SARS-CoV-2, VOC

## Abstract

The emergence of the SARS-CoV-2 B.1.617.2 lineage (Delta variant) in 2021 was associated with increased case numbers and test positivity rates, including a large number of infections in fully vaccinated individuals. Here, we describe the findings of an investigation conducted in Tompkins County, New York, to evaluate factors underlying a significant uptick in the number of coronavirus disease 2019 (COVID-19) cases observed in the months of July and August 2021. We performed genomic surveillance and genotyping as well as virological assessments to determine infectivity of the virus in a select number of clinical diagnostic samples. Genomic sequence analyses revealed complete replacement of the B.1.1.7 lineage (Alpha variant) with the B.1.617.2 lineage (Delta variant) between July 1 and August 4 2021. We observed a strong association between viral RNA loads detected by real-time reverse transcriptase PCR and infectious virus detected in respiratory secretions by virus titration. A marked increase in positive cases among fully vaccinated individuals was observed. The sequence divergence between two index Delta variant cases in April and May, and the cases after July 1st, revealed independent Delta variant introductions in Tompkins County. Contact tracing information enabled the detection of clusters of connected cases within closely related phylogenetic clusters. We also found evidence of transmission between vaccinated individuals and between vaccinated and unvaccinated individuals. This was confirmed by detection and isolation of infectious virus from a group of individuals within epidemiologically connected transmission clusters, confirming shedding of high viral loads and transmission of the virus by fully vaccinated individuals.

**IMPORTANCE** The SARS-CoV-2 lineage B.1.617.2 (Delta variant) emerged in Asia and rapidly spread to other countries, becoming the dominant circulating lineage. Worldwide infections with B.1.617.2 peaked at a time in which vaccination rates were increasing. In this study, we present data characterizing the emergence of SARS-CoV-2 lineage B.1.617.2 (Delta variant) in Tompkins County, New York, which has one of the highest vaccination rates in the state. We present evidence demonstrating infection, replication, and transmission of SARS-CoV-2 lineage B.1.617.2 (Delta variant) between fully vaccinated individuals. Importantly, infectious virus loads were determined in a subset of samples and demonstrated shedding of high viral titers in respiratory secretions of vaccinated individuals.

## INTRODUCTION

Severe acute respiratory syndrome coronavirus 2 (SARS-CoV-2) has caused more than 360 million infections and over 5.6 million deaths worldwide since the first detection of the virus in Wuhan, China in December 2019 (https://covid19.who.int/). One of the hallmarks that contributed to the rapid spread of SARS-CoV-2 and to an unprecedented number of infections globally is the high transmissibility of the virus. Herculean efforts from governments, academia, and industry led to the rapid development and deployment of several coronavirus disease 2019 (COVID-19) vaccines. The high efficacy demonstrated by these vaccines in clinical trials (up to 95%) led to their rapid approval under emergency use authorizations. Notably, the clinical use of these vaccines led to a significant decline in the number of COVID-19 cases in nearly all countries in which they were deployed.

The emergence of SARS-CoV-2 variants containing mutations in key amino acid residues in the spike (S) protein, the main target of current COVID-19 vaccines, poses some challenges for the continued efficacy of vaccines and of monoclonal antibody-based therapeutics. The emergence of the SARS-CoV-2 B.1.617.2 lineage (Delta variant) has been associated with increased case numbers and test positivity rates, including a large number of infections in fully vaccinated individuals (1–3), suggesting rapid community spread and potential escape from vaccine-induced immunity. Infections with the B.1.617.2 lineage (Delta) have also been associated with higher viral loads (1, 2, 4, 5) and increased transmissibility compared to other SARS-CoV-2 variants (5, 6). Thus, it is critical to understand the factors leading to SARS-CoV-2 B.1.617.2 lineage (Delta) spread in areas where vaccination coverage is high. Most importantly, it is essential to determine whether vaccinated individuals who become infected can shed and transmit the virus to others. This information has important public health implications and may help authorities to devise new and/or refined recommendations for effective control of the COVID-19 pandemic.

In the present study, we evaluated the findings of an investigation conducted in Tompkins County, New York, to identify factors underlying a significant spike in the number of COVID-19 cases observed in the months of July and August 2021. Tompkins County has a population of approximately 106,000 people and is one of the counties with the highest COVID-19 vaccination coverage in the state of New York, with approximately 65% of the population being fully vaccinated and 69% being vaccinated with at least one dose by August 2021. We performed genomic sequencing and genotyping as well as virological assessments to determine infectivity of the virus in a select number of genetically and epidemiologically related clusters.

## RESULTS

### Increased cases of COVID-19 in Tompkins County during the summer of 2021.

The Tompkins County Health Department followed the New York guidance in rolling out COVID-19 vaccines, with the first doses being administered to health care workers in December 2020. Vaccines were then progressively rolled out to elderly people, individuals with underlying health conditions, essential workers, and finally the general population. By April 2021, anyone aged 16 years or older was eligible to be vaccinated, and those aged 12 to 15 years became eligible in May 2021.

**FIG 1:**
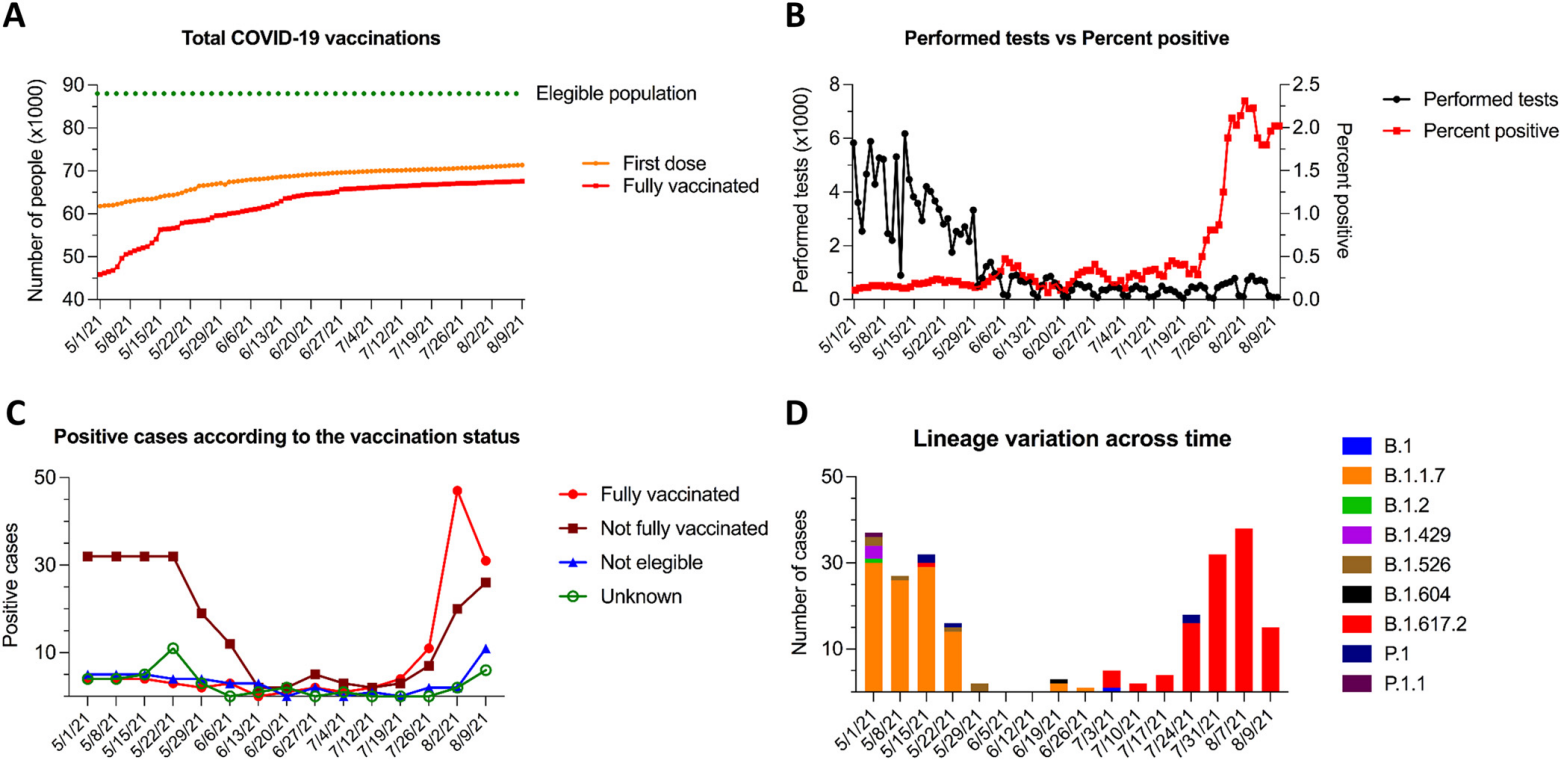
COVID-19 vaccinations rates, SARS-CoV-2 rRT-PCR results, and viral lineages detected in Tompkins County, NY, from 1 May to 9 August 2021. (A) Number of COVID-19 vaccinations. Fully vaccinated was defined as individual received two vaccine doses in a 2-dose vaccine series or one dose of a single-dose vaccine. (B) SARS-CoV-2 rRT-PCR test results and the percentages of positive results. (C) Positive cases of COVID-19 according to individual vaccination status. “Not fully vaccinated” are unvaccinated individuals, those who had not completed the 2-dose series, or those who had a COVID-19-positive test earlier than 2 weeks after their second vaccine dose. “Not eligible” are individuals under age 12 who were not eligible for the vaccine at the time of the study. “Unknown” are individuals for whom vaccination status could not be confirmed. (D) SARS-CoV-2 lineages identified from 1 May to 9 August 2021 in Tompkins County, NY. The population of Tompkins County was estimated at 102,180 in 2019.

By the start date of the study (May 1 2021), ~62% of Tompkins County’s population had received at least one dose of a COVID-19 vaccine, and ~46% of the population was fully vaccinated (defined as 2 weeks after they had received the second dose in a 2-dose vaccine series or at least 2 weeks after they had received a single-dose vaccine). By the end of the study period (August 9 2021), the proportion of the Tompkins County population that was fully vaccinated had reached ~60% (Fig. 1A).

### SARS-CoV-2 infections and variant dynamics over time.

Genomic surveillance data revealed complete replacement of the B.1.1.7 lineage (Alpha) with the B.1.617.2 lineage (Delta) between July 1 and July 3 (111 of 118 cases, 94%). Before this shift, other SARS-CoV-2 lineages, including B.1, B.1.2, B.1.429, B.1.526, B.1.604, P.1, and P.1.1, were detected in the county, albeit in reduced numbers (Fig. 1D and Table 1).

**TABLE 1:** Characteristics of individuals with SARS-CoV-2 infection during emergence of the B1.617.2 (Delta) variant

Variable	Result^*a*^
Total no. of individuals COVID-19-positive by rRT-PCR (July 1 to August 4 2019)	118
Vaccination status	47 fully vaccinated, 25 unvaccinated, 46 unknown status
Vaccine brand	22/47 Moderna, 22/47 Pfizer, 3/47 Janssen
Time since fully vaccinated and positive rRT-PCR (avg ± SD)	110 ± 32 days (NA = 10)
Age (avg ± SD)	36.7 ± 20.1 years (NA = 8)
Sex	57 F, 53 M (NA = 8)
Symptoms	72/76 (95%) symptomatic, 4/76 (5%) asymptomatic (NA = 42)
Symptoms among fully vaccinated individuals	44/46 (96%) symptomatic, 2/46 (4%) asymptomatic (NA = 1)
Symptoms among unvaccinated individuals	17/17 (100%) symptomatic (NA = 8)
Hospitalization	5 individuals, of whom 4 were unvaccinated (NA = 1)
Death	2 unvaccinated individuals
Virus lineages, no. (%)	B.1 = 1 (0.8%), B.1.1.7 = 3 (2.6%); B.1.604 = 1 (0.8%); B1.617.2 = 111 (94%); P.1 = 2 (1.7%)

a NA, data not available.

After almost a month with a low number of cases (<5 per day), we observed an inverse correlation between the number of tests performed and the percent positivity rate (Fig. 1B). The number of performed tests decreased substantially at the end of May, while the percentage of positive cases remained low until mid-July, with a marked increase observed after the second week of July (Fig. 1B). Notably, increased numbers of SARS-CoV-2 infections were associated with the emergence of the B.1.617.2 lineage (Delta) in the county (Fig. 1B, C, and D).

### Vaccine breakthrough infections and viral loads.

At the end of June, more than 60% of the Tompkins County population was fully vaccinated (Fig. 1A), and the number of SARS-CoV-2 cases was very low (<5 cases per day) (Fig. 1C). Approximately 1 month later, a peak of SARS-CoV-2 cases was observed in the county, with most of the cases occurring in fully vaccinated individuals (Fig. 1C). Among fully vaccinated individuals, 96% of cases were mildly symptomatic (Table 1), while 100% of cases among unvaccinated individuals were symptomatic. From a total of 118 positive individuals, 47 were fully vaccinated with an average of 110 days between the second vaccine dose and the positive test and detection of Delta variant infection. Importantly, an equal number of individuals (*n* = 22) were vaccinated with Moderna or Pfizer vaccines, while 3 people that tested positive were vaccinated with the Janssen vaccine. It is important to note that the Moderna and Pfizer vaccines were the main vaccines being used in Tompkins County.

**FIG 2:**
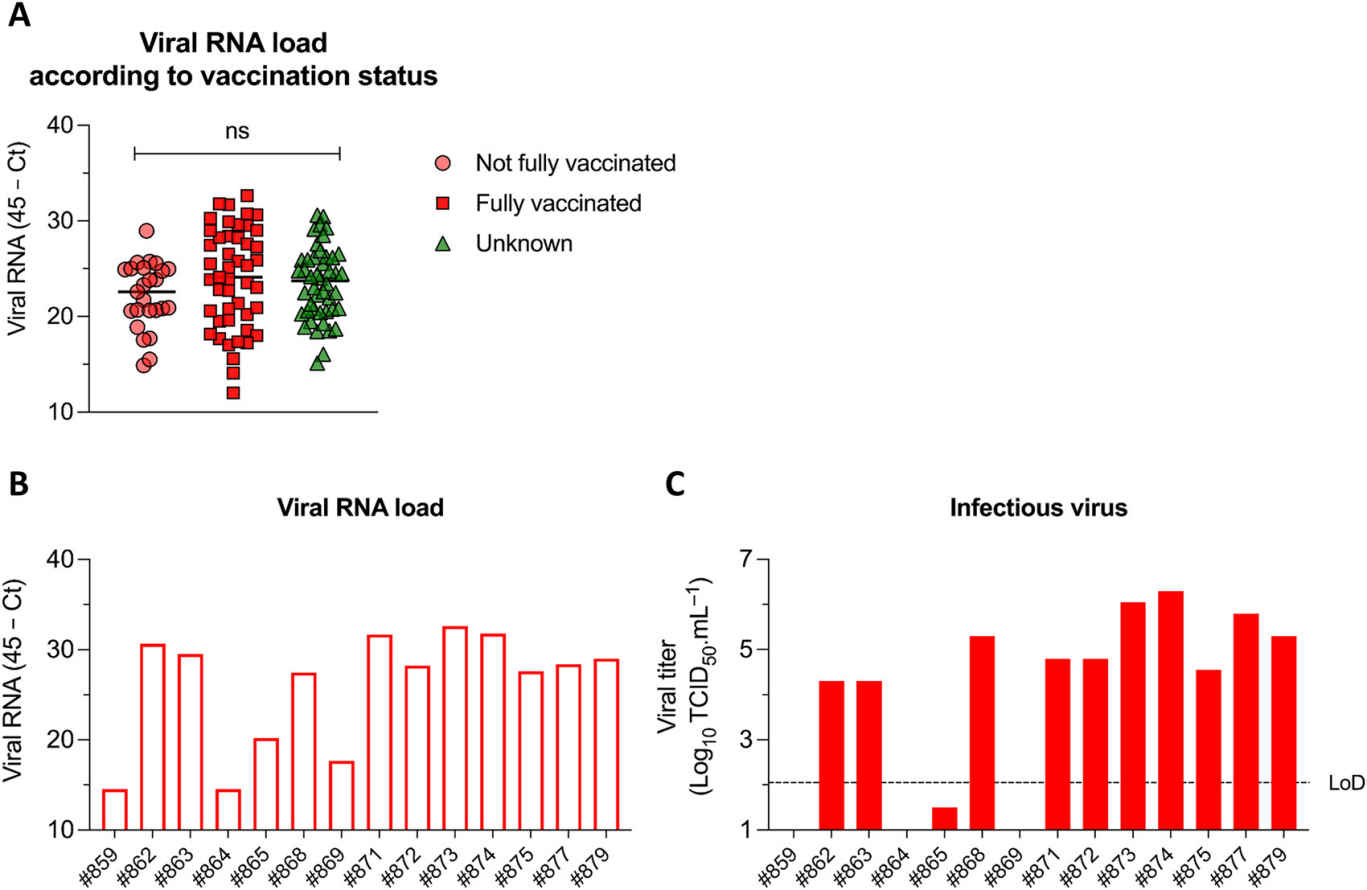
Viral RNA load according to vaccination status and infectious virus titers in vaccinated individuals. (A) SARS-CoV-2 RNA load detected by rRT-PCR according to vaccination status. “Not fully vaccinated” are unvaccinated individuals, those who had not completed the 2-dose series, or those who had a COVID-19-positive test earlier than 2 weeks after their second vaccine dose. “Unknown” are individuals for whom vaccination status could not be confirmed. (B) SARS-CoV-2 RNA load in a subset of anterior nares (AN) swabs collected from 14 fully vaccinated individuals and that were conditioned in viral transport medium without an inactivating solution. (C) Infectious SARS-CoV-2 titers in the same subset of AN swabs collected from 14 fully vaccinated individuals and that were conditioned in viral transport medium without an inactivating solution. Virus titers were determined based on fluorescence-positive wells, using the Spearman and Karber method, and are expressed as the TCID_50_ per milliliter.

The real-time reverse transcriptase PCR (rRT-PCR) threshold cycle (*C_T_*) values for unvaccinated, fully vaccinated, and individuals with an unknown vaccination status were compared. Interestingly, no significant differences in *C_T_* values were observed between fully vaccinated and unvaccinated individuals (Fig. 2A). To assess shedding of infectious virus, a subset of samples with the Delta variant, collected from fully vaccinated individuals (*n* = 14), were conditioned in viral transport medium without an inactivating solution and subjected to virus isolation and titrations. High viral RNA loads were observed in those samples (Fig. 2B), and, more importantly, infectious SARS-CoV-2 was recovered from 11 of the 14 samples, with 10 of the 14 individuals shedding high infectious virus titers, ranging from 10^4^ to 10^6.8^ 50% tissue culture infective doses (TCID_50_)/mL (Fig. 2C).

### Evidence of SARS-CoV-2 B.1.617.2 lineage (Delta variant) transmission among vaccinated individuals.

**FIG 3:**
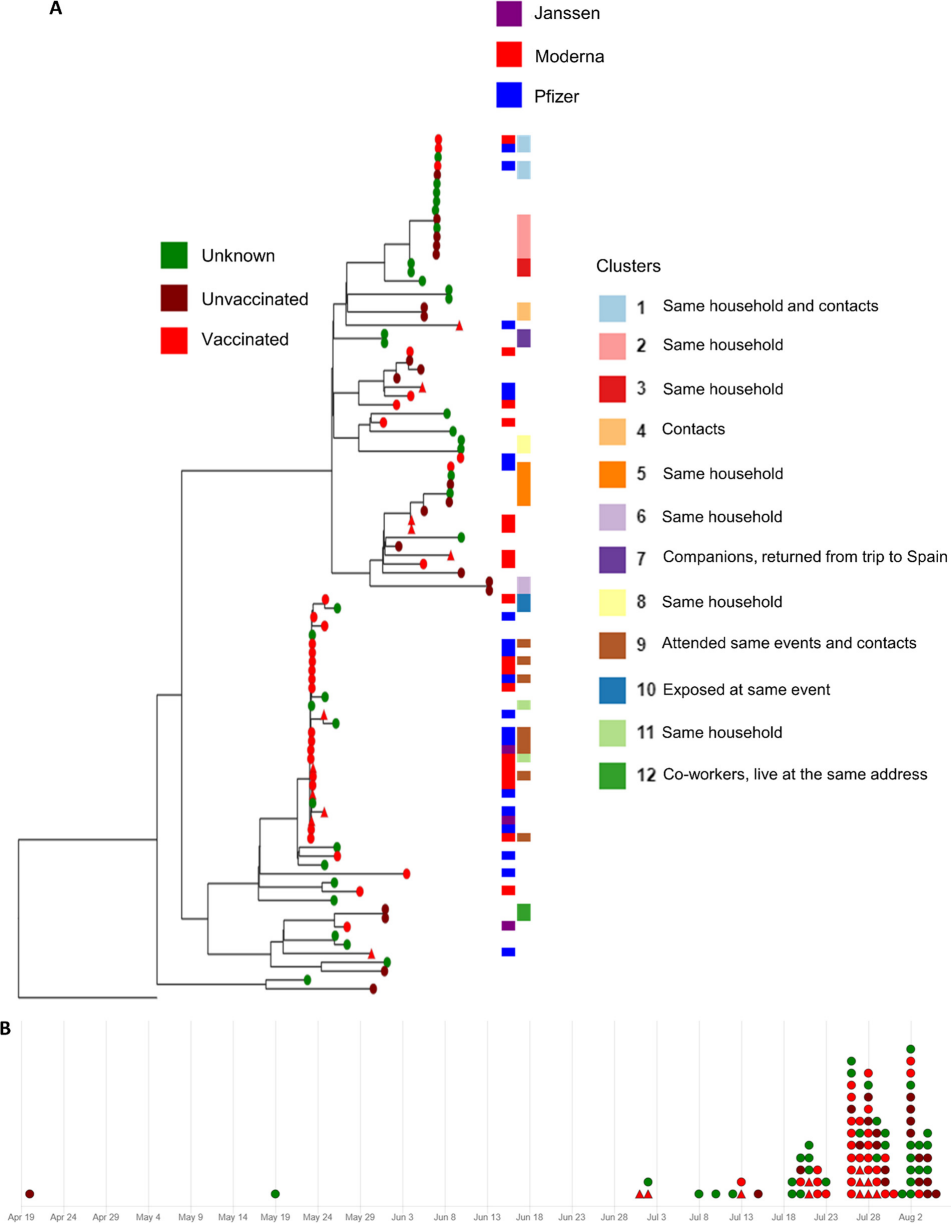
Phylogenetic tree and timeline of sequences classified as Delta lineage, detected in Tompkins County, NY, from May 1 to August 9 2021. (A) Nodes in the phylogenetic tree are differentiated by colors representing vaccination status. Nodes with a triangular shape represent samples that were successfully isolated in cell culture. The first column of metadata blocks represents the vaccine brand administered in the individuals with vaccinated status in the phylogenetic tree nodes. The second column of metadata blocks characterizes individuals by epidemiological clusters, differentiated by colors. (B) Timeline panel of detections. All the sequences used to build the phylogenetic tree are represented in the timeline, on the respective date of detection. Each circle or triangle represents an individual.

To assess the genetic makeup and determine potential genomic link between epidemiological clusters of SARS-CoV-2, a phylogenetic analysis using complete SARS-CoV-2 sequences was conducted. The sequence divergence between the index B.1.617.2 lineage (Delta variant) cases and the other B.1.617.2 sequences detected after July 1 revealed independent Delta variant introductions in Tompkins County. As shown in Fig. 3, two major genetic clusters of B.1.617.2 sequences were detected. One of these clusters was less diverse, and it was composed mostly of B.1.617.2 sequences recovered from vaccinated individuals (Fig. 3, bottom cluster in the tree). Eight fully vaccinated individuals within that cluster were identified as a group of friends who attended several social gatherings together over a period of a week. Additionally, several other small sequence clusters and matching epidemiological links (e.g., same household, coworkers, travel) were observed (Fig. 2). These smaller clusters were characterized by individuals with a different vaccination status (vaccinated, unvaccinated, or unknown). The sequence analyses combined with epidemiological and contact tracing information revealed likely transmission of SARS-CoV-2 B.1.617.2 lineage (Delta variant) between fully vaccinated individuals. It was not possible to detect any relation between vaccine manufacturer and phylogenetic cluster, i.e., there was no relation between vaccine type and molecular characteristics of strains presented. Three mutations were found in the spike protein with frequencies ranging from 22.4 to 31.6% within the 98 sequences analyzed: a T-to-I substitution at position 95 (T95I), A222V, and V1264L. These mutations were found in sequences recovered from both vaccinated and nonvaccinated individuals. Analyses of genomic sequence data with vaccination status allowed us to identify evidence of transmission of the B.1.617.2 lineage between vaccinated and unvaccinated individuals which, when considered in the context of viral loads being shed by vaccinated individuals (Fig. 2C), was not a complete surprise.

## DISCUSSION

Genomic surveillance for SARS-CoV-2 performed in Tompkins County, NY, revealed complete replacement of the B.1.1.7 lineage (Alpha variant) with the B.1.617.2 lineage (Delta) after July 1 2021. It was not possible to trace a unique index case, given the close genetic relationships between the sequences detected. Although two B.1.617.2 lineage cases were first detected in April and May, phylogenetic analysis suggested that these two cases did not result in community transmission. Analyses of the sequences recovered after July 1 2021 revealed multiple introductions of the B.1.617.2 lineage (Delta variant) in the county. We were able to detect multiple intralineage mutations among the sequenced samples. Interestingly, the mutation V1264L, detected in some of our sequences, was present only in three Delta sublineages and also lineage AB.1 (7). None of the sequences detected in our study harbored the K417N mutation, which is associated with escape from neutralization (8) and is found in certain Delta sublineages. A recent study revealed, however, that Delta variant sublineages do not show increased neutralization resistance (9).

Although *C_T_* values do not provide absolute virus quantification, we observed an association with viral titers (Fig. 2B and C). Samples with *C_T_* values lower than 30 were successfully isolated in cell culture, consistent with a high load of infectious virus (10, 11). High viral loads detected in fully vaccinated individuals likely played an important role in the successful transmission of the virus. Interestingly, while before the emergence of the B.1.617.2 lineage in Tompkins County the number of cases in fully vaccinated individuals was very low, once the variant was detected a spike in cases was observed. Our findings demonstrating shedding of infectious virus by vaccinated individuals are consistent with other reports (1, 2, 12); however, the high viral loads detected here (shedding of infectious virus titers ranging from 10^4^ to 10^6.8^ TCID_50_/ml) were somewhat surprising. The effectiveness of the Pfizer vaccine against symptomatic disease has been shown to decline with time after vaccination (13), despite protecting against severe disease and death (14). Similarly, mild symptoms were observed in the individuals that tested positive in our study, with 96% of fully vaccinated individuals presenting symptoms. Importantly, the only two individuals that were hospitalized and died of the infection were not vaccinated. The average time between the second vaccine dose and the positive rRT-PCR result and detection of the B.1.617.2 lineage was 110 days. Although for most vaccines this would be considered a short period our data suggest that for COVID-19 vaccines this time frame may represent the period in which vaccine-elicited immunity might decline. The replication capability of SARS-Co-V-2 variants such as the B.1.617.2 lineage (Delta) suggest higher infectiousness during the early stage of infection (1). This could facilitate transmission of the virus. A higher proportion of cleaved spike protein of B.1.617.2 live virus compared to B.1.1.7 was suggested to be involved in the mechanism of increased infectivity through enhanced virus entry into a range of target cells (4). The increased replication capability of the Delta variant, together with a decline in circulating antibodies, may explain the lack of differences in viral loads between vaccinated and unvaccinated individuals.

### Conclusion.

Our data suggest that frequent COVID-19 vaccine boosters might be required to increase the level of immunity and breadth of protection provided against variant SARS-CoV-2 variants that may emerge in the future. Notably, the recent emergence of the B.1.1.529 lineage (Omicron variant) reinforces this notion. Our data also underscore the power of large-scale whole-genome sequencing for detection of mutations or rapid characterization of newly emerging variants, combined, to establish epidemiologic connections between positive SARS-CoV-2 cases.

## MATERIALS AND METHODS

### Study design and samples.

In this retrospective cohort study, we performed genomic surveillance in deidentified diagnostic samples (*n* = 118) and analyzed demographic records from Cayuga Health System (CHS) and Tompkins County Health Department (Ithaca, NY, USA) to understand the underlying factors associated with an increase in the number of COVID-19 cases in fully vaccinated individuals observed from July to August 2021. The study population consisted of all CHS patients with a positive diagnosis of COVID-19. The study period (May 1 2021 to August 9 2021) corresponded to a period in which a marked increase in positive COVID-19 cases was observed in Tompkins County.

Genomic surveillance was conducted in remnant specimens used for initial COVID-19 diagnosis at CHS and included deidentified diagnostic samples from the broader Tompkins County community and the Cornell University public health surveillance program.

### Ethics statement.

The work was reviewed and approved by the relevant institutional review boards (IRBs) at Cayuga Health System (protocol 0420EP) and Cornell University (protocol 2101010049).

### Vaccination status definitions.

Any COVID-19 vaccines administered in Tompkins County are captured using the Tompkins County Health Department COVID-19 Vaccine Registry. Individuals were considered “fully vaccinated” 2 weeks after they received the second dose of a 2-dose vaccine series or 2 weeks after they had been vaccinated with a single-dose vaccine. The category “not fully vaccinated” comprised unvaccinated individuals or those who had not completed the 2-dose series or that had a COVID-19 positive test earlier than 2 weeks after their second vaccine dose. Individuals under age 12 who were not eligible for the vaccine at the time of the study were categorized as “not eligible,” while individuals in which the vaccination status could not be confirmed were included in the “unknown” category. In total, this study comprised 47 fully vaccinated individuals, 25 not fully vaccinated, and 46 unknown.

### Clinical definitions, specimens, and testing outcomes.

Testing results performed in Tompkins County between May 1 and August 9 2021 were included in our study. Any sample testing positive for SARS-CoV-2 via rRT-PCR performed in any clinical specimen (nasopharyngeal or anterior nares swabs or saliva), regardless of the presence of symptoms, was included. COVID-19-related hospital admissions were defined as a patient admitted with a positive SARS-CoV-2 RT-PCR test within 14 days of admission.

SARS-CoV-2 rRT-PCR-positive samples with *C_T_* values of <30 were processed for whole-genome sequencing and lineage classification. A subset of positive samples of genetically and epidemiologically related clusters, which were collected in viral transport medium without inactivating medium, were subjected to virus isolation and quantitation via titrations in cell culture as described below.

### Whole-genome sequencing.

Whole-genome sequencing was performed directly in nucleic acids from clinical specimens by using the MinION (ONT) with multiplexed tiled amplicon approach, as previously described (15). Reads were processed through the ARTIC ncov-2019 bioinformatic pipeline, using Medaka for variant calling (https://artic.network/ncov-2019/ncov2019-bioinformatics-sop.html). The pipeline assigns an N value to bases with <20× read depth. All consensus genomes were deposited in the Global Initiative on Sharing Avian Influenza data (GISAID) and in GenBank (see “Data availability,” below, for details).

### Variant identification and phylogenetic analysis.

Viral lineage designations and classifications were performed using Pangolin v3.1.11 (https://github.com/hCoV-2019/pangolin). Sequences with more than 70% of called bases classified as the Delta variant were used for a whole-genome nucleotide alignment, performed using MAFFT v7.450 (16). A maximum-likelihood tree was constructed using IQ-tree v1.6.12 (17) by applying the GTR+F+I model of nucleotide substitution and 1,000 bootstrap replicates. The tree was rooted at the Wuhan-1 reference strain (NC_045512.2). The Newick format of phylogenetic trees and a metadata table were used as input for visualization of the tree and timeline in MicroReact (18). A cutoff of 3% was set as the threshold for minor allele frequency, to exclude potential PCR and sequencing errors.

### Virus isolation and viral loads.

Anterior nares swab samples, which were conditioned in viral transport medium, were subjected to virus isolation under biosafety level 3 (BSL-3) conditions at the Cornell Animal Health Diagnostic Center BSL-3 research suite. For this, six-well plates were seeded with ~300,000 Vero E6/TMPRSS2 cells per well, 24 h prior to sample inoculation. Cells were rinsed with phosphate-buffered saline (PBS; Corning) and inoculated with 200 μL of each sample (swab supernatant) in addition to 400 μL of Dulbecco's modified Eagle's medium (DMEM), and the inoculum adsorbed for 1 h at 37°C with 5% CO_2_. Mock-inoculated cells were used as negative controls. After adsorption, cells were rinsed with PBS and DMEM was replaced, supplemented with 10% fetal bovine serum, l-glutamine (2 mM), penicillin (100 U/mL), streptomycin (100 μg/mL), and gentamicin (50 μg/mL), and cells were incubated at 37°C with 5% CO_2_ and monitored daily for viral cytopathic effect (CPE) for 3 days. Cell cultures with no CPE were frozen, thawed, and subjected to two additional 3-day blind passages in Vero E6/TMPRSS2 cell cultures.

Virus isolation-positive samples were subjected to endpoint titrations by limiting dilution using Vero E6/TMPRSS2 cells. For this, the original samples were serially diluted in DMEM (10-fold dilutions from 10^−1^ to 10^−6^), and individual dilutions were inoculated into Vero E6/TMPRSS2 cultures in 96-well plates prepared 24 h in advance. The cells were incubated at 37°C with 5% for 48 h and then fixed with 3.7% formaldehyde for 30 min at room temperature. After fixation, the cells were permeabilized with 0.2% Triton X-100 (in PBS) for 10 min at room temperature and subjected to immunofluorescence assay (IFA) using a monoclonal antibody, anti-SARS-CoV-2 nucleoprotein (N; clone B61G11, developed in our laboratory) during 1 h at 37°C, followed by 1 h of incubation at 37°C with a goat anti-mouse IgG (with Alexa Fluor 594 label). Virus titers were determined based on fluorescence-positive wells using the Spearman and Karber method and expressed as the TCID_50_ per milliliter.

### Data availability.

All consensus genomes were deposited in GISAID (https://gisaid.org/) under accession numbers EPI_ISL_5903278 to EPI_ISL_5999262 and in GenBank under accession numbers OM470973 to OM471069.
